# Video- Versus Text-Based Psychoeducation in Web-Based E-Mental Health Programs: Randomized Controlled Trial

**DOI:** 10.2196/65478

**Published:** 2025-06-24

**Authors:** Swantje Borsutzky, Josefine Gehlenborg, Lara Rolvien, Steffen Moritz

**Affiliations:** 1 Department of Psychosocial Medicine University Hospital Hamburg-Eppendorf Clinic for Psychiatry and Psychotherapy Hamburg Germany

**Keywords:** e-mental health, psychoeducation, knowledge gain, unguided interventions, sleep disorders, social competence deficits, confidence in learning, experimental conditions, transdiagnostic interventions

## Abstract

**Background:**

Mental health disorders affect 1 in 8 people worldwide, yet many face barriers to accessing care. E-mental health interventions, including self-guided internet-based programs, offer promising solutions. However, the mechanisms driving knowledge gain in such programs remain poorly understood. The role of medium, topic, sequence, and confidence and their interaction in learning outcomes need further investigation. Additionally, the influence of knowledge gaps on the outcome of psychoeducational intervention is not well understood (eg, whether psychoeducation requires an existing knowledge gap to be effective).

**Objective:**

This randomized controlled trial investigated the role of medium, topic, sequence, and participants’ initial knowledge levels on knowledge gain and confidence in fully automated self-guided e-mental health psychoeducation.

**Methods:**

A total of 158 adults (mean age 34, SD 12.4 years; n=118, 74.7% female) were randomized to 8 experimental conditions (receiving video, texts, or both containing psychoeducational content on sleep or social competence; n=142) or a control group (neutral video; n=16). The fully automated interventions (videos) were developed for use in web-based e-mental health interventions. They address transdiagnostic symptoms and hence are relevant across various disorders. To assess the added value of video production for knowledge gain, text-based scripts corresponding to the video content were created and compared. All interventions and outcome assessments were delivered on the web via Qualtrics without face-to-face components. Pre- and postintervention knowledge was assessed using a validated 30-item knowledge test (true/false). Confidence in responses was rated on a 0% to 100% scale. Statistical analyses included 3-way ANOVA and multivariate ANOVA.

**Results:**

Knowledge significantly increased across experimental groups (*F*_1,156_=17.272; *P*<.001; η_p_^2^=0.10). Participants with social competence deficits had significantly lower baseline knowledge (*P*=.04; *d*=0.41). For sleep deficits, a nonsignificant trend emerged (*P*=.09; *d*=0.28). Participants with social competence deficits demonstrated greater knowledge improvement (t_141_=7.12; *P*<.001; *d*=0.60). Participants with sleep deficits showed smaller but significant gains (t_141_=2.43; *P*=.02; *d*=0.20). No significant differences in knowledge gain were found between video and text formats. Confidence in correct answers increased significantly in the experimental group (mean 42.82, 95% CI 41.15-44.50 to mean 51.67, 95% CI 49.28-54.04), with larger gains for social competence than sleep. Confidence in the control group remained unchanged.

**Conclusions:**

Both video and text formats effectively facilitated knowledge gain in e-mental health interventions, with no clear advantage of one medium over the other. Participants with prior deficits learned more in areas where they initially lacked knowledge. Confidence in correct answers increased alongside knowledge, highlighting psychoeducation’s role in promoting self-efficacy. Future research should explore multimedia integration to enhance adherence and symptom improvement.

**Trial Registration:**

German Clinical Trials Register DRKS00026722; https://drks.de/search/en/trial/DRKS00026722

## Introduction

Mental health disorders affect 1 in 8 people worldwide, with depression and anxiety being the most common conditions [1]. Notwithstanding effective prevention and treatment options (eg, cognitive behavioral therapy or medication), one-third of people with depression worldwide lack access to care, according to the World Health Organization [2]. Reasons for this treatment gap range from structural barriers (eg, long waiting lists to receive therapy due to a lack of psychotherapists, being in a remote location, or facing financial barriers) to personal factors (eg, fear of stigmatization, negative health beliefs, or a lack of awareness of treatment options) [3]. Failing to provide adequate treatment for mental disorders not only worsens the condition but also increases societal and economic costs [3], highlighting the urgent need for access to support. In response to this need, alternatives to traditional face-to-face psychotherapy, such as self-guided e-mental health programs, are gaining attention in mental health care. These programs offer flexibility and anonymity and therefore appeal to a broad audience [4,5]. Many self-guided e-mental health programs are based on psychoeducation, which involves providing structured information about a patient’s disorder and treatment options. The term *psychoeducation* was first used by Anderson and colleagues [6] in a manual on schizophrenia that aimed to empower patients and their families by enhancing their understanding of the illness and improving their coping strategies [7]. Psychoeducation today covers various topics, from improving sleep to social competence skills, and it has shown promising results across different mental disorders, according to meta-analyses and literature reviews [8-13]. Transdiagnostic problems like sleep problems and poor social competence are particularly relevant in e-mental health programs, as they address symptoms that are common across multiple mental health disorders [14].

Knowledge gain as part of psychoeducation is essential in successful treatment because well-informed patients tend to benefit more from therapy [15]. In self-guided e-mental health interventions, which lack the direct interaction with therapists of face-to-face psychotherapy, adequate knowledge gain may not occur, thus potentially compromising treatment outcomes. So far, only a few studies have examined the mechanisms of knowledge gain that may lead to better therapy outcomes in face-to-face psychotherapeutic settings. Previous research [14] suggests enhancing the recall of therapy content to increase the effectiveness of psychoeducational interventions. Abramowitz and colleagues [16] found that patients with obsessive-compulsive disorder who received psychoeducation prior to psychotherapy better understood the rationale and were more compliant, suggesting that knowledge gain is a powerful mechanism of change in psychotherapy. Abramowitz and colleagues [16] suggested using quizzes to test knowledge and assumed that quantity (eg, attending all therapy sessions) would be less important than gaining knowledge about the treatment rationale. However, the extent of patients’ knowledge about their symptoms generally and prior to therapy remains unclear and requires further investigation.

Research on methods of knowledge gain in therapeutic settings is minimal. In educational settings, effective ways to convey knowledge have been studied for decades. A meta-analysis of 51 studies found that students who took online classes outperformed students receiving face-to-face instruction, with an average effect size that was small (*d*=0.24) [17]. Moreover, online learning has no time or location limitations and thus increases accessibility. Recent meta-analytic evidence further supports these findings. For example, a meta-analysis by Ebner and Gegenfurtner [18] demonstrated that online learning formats, including webinars, can achieve comparable or even superior outcomes in terms of learning effectiveness when compared to traditional face-to-face formats. Another recent meta-analysis found that instructional in-class videos in educational contexts achieved stronger learning effects (*d*=0.69) than other formats such as outside-class videos, texts, or video games [19].

Two prevalent media for conveying knowledge in e-mental health interventions are videos and texts. In the framework of e-learning for clinical procedures, videos were more effective for learning outcomes than illustrated, text-based online instructions in a sample of medical students [20]. However, the literature on optimal media for knowledge gain in e-mental health programs is limited. Addressing this gap, Schlarb and colleagues [21] explored in their pilot study the acceptance and feasibility of online prevention and early intervention training for university staff with self-reported sleep issues. Participants found the videos helpful and entertaining and had improved insomnia symptoms with medium effect sizes. Further research suggests that, compared to text, information provided via video is more engaging and enhances content retention [22].

Researchers have also investigated judgment certainty, which is another moderator of knowledge gain. As early as 1977, Fischhoff and colleagues [23] examined people’s tendency to be overconfident concerning their knowledge. Subsequent studies, such as one by Martí [24], have linked certainty to learners’ performance and feedback. However, in educational settings, Dory and colleagues [25] found that medical students’ confidence levels were not necessarily correlated with their test performance. To date, the role of judgment certainty on knowledge gain in clinical settings remains largely unexplored.

### Objectives

One aim of this research project was to understand moderators of success in knowledge gain. Specifically, this study explored the role of the medium (text or video), topic (sleep or social competence), and sequence of presentation (ie, whether the medium changed or remained the same, that is, video to text vs text to text) in knowledge gain in low-threshold online experimental conditions targeting the topics of sleep and social competence. We also aimed to examine whether participants with deficits related to sleep and/or social competence had knowledge gaps in these areas and whether this could be compensated for through targeted psychoeducation. Finally, we investigated whether knowledge gain would lead to an increase in high-confidence correct judgments and/or a decrease in overconfidence in incorrect judgments.

## Methods

### Participants

A total of 158 adults with a mean age of 34 (SD 12.4) years participated in the study. Most participants were female (n=118, 74.7%); the majority (n=88, 55.5%) had an education level above a high school degree.

To analyze knowledge gaps and improvements, participants were divided into 2 groups based on reported deficits: those with subjective social competence deficits (n=21) and those with subjective sleep deficits (n=48). Social competence deficits were defined as scoring below 60% on a self-report scale (0% to 100%); sleep deficits were based on a binary “yes” or “no” self-report. “Yes” responses assigned participants to the sleep deficit group. Participants were randomly assigned to 1 of 8 experimental conditions or a control group (neutral videos: n=16), as described in the Procedure section.

### Sample Size and Power Calculation

A priori power analysis was conducted using G*Power (version 3.1; Heinrich-Heine-Universität) to determine the minimum sample size required for the study. Assuming a small to medium effect size (*f*=0.25), an α level of .05, and a power of 0.80, the estimated sample size required for a 3-way ANOVA with repeated measures (eg, time, topic, and condition) was 156 participants. This calculation ensured sufficient statistical power to detect meaningful differences between the experimental and control conditions across time and topics. The recruited sample size of 158 participants met this requirement.

### Ethical Considerations

#### Ethics Approval and Trial Registration

The study was approved by the ethics committee of the University Medical Center Hamburg-Eppendorf, Germany (LPEK-0399) and was conducted in accordance with the Declaration of Helsinki. This study is part of a larger research project aimed at developing and evaluating an e-mental health intervention. Sleep and social competence represent two key modules within the program. The interventions examined in this trial were specifically designed as integral components of this broader program. The trial is registered in the German Clinical Trials Registration under the identifier DRKS00026722. This is a pilot trial informing a larger and independent randomized controlled trial that has not yet been published.

#### Informed Consent

All participants provided electronic informed consent before beginning the study. They were informed about the purpose and procedures, as well as their right to withdraw at any time without consequences. No waiver of consent was applied.

#### Privacy and Confidentiality

The data collected in this study were anonymized and stored in compliance with the General Data Protection Regulation. No personally identifiable information was collected or retained, and all data were analyzed in aggregated form to ensure confidentiality.

#### Compensation

Participants received access to the COGITO (www.uke.de/cogito) self-help smartphone app as an incentive for their participation.

### Procedure

Participants were recruited online through Facebook groups and Facebook pages focused on mental health, well-being, and psychology (eg, forums for self-help and personal development). Recruitment posts included a brief explanation of the study purpose, eligibility criteria (eg, being aged 18 to 75 years, having internet access, and being proficient in German), and a link to the survey on the Qualtrics platform. The recruitment messages emphasized the voluntary nature of participation and informed potential participants about the incentive for completing the study—access to the COGITO self-help app.

Individuals reporting suicidal ideation, assessed using item 15 of the Web Screening Questionnaire [25], were excluded to ensure participant safety. The recruitment process aimed to reach a broad audience. The study was conducted online without face-to-face assessment. After providing informed consent, participants completed questions on demographic characteristics as well as questions on sleep and social competence. A baseline knowledge test followed. The knowledge test was designed specifically for this study and was reviewed by experts to ensure content validity. Participants were then randomly assigned to 1 of 9 experimental or control conditions using Qualtrics software, ensuring balanced group sizes. The study used a parallel-group randomized controlled trial design. There were no participant dropouts during the study, and all randomized participants completed the interventions. No changes to the study methods, intervention materials, or randomization procedures were made after the trial commenced.

Experimental groups received videos, texts, or both containing content on both topics (sleep and social competence), while the control group watched a video on a neutral topic unrelated to sleep or social competence (a tutorial on using a self-help app). To assess whether the order of the media might be a mechanism in knowledge gain, the order of text versus videos varied between experimental conditions. To prevent participants from skipping information by continuing without watching a video or reading a text, a timer was added. After the timer had expired (after 2.5 minutes), participants could continue the program. After presentation of the learning materials or of neutral stimuli in the control group, the postintervention knowledge test was administered. Blinding was not possible in this study due to the nature of the interventions, as participants were aware of whether they were receiving video or text materials. However, assessments were conducted as planned after randomization, and participants were not fully informed about the specific hypotheses of the study.

### Outcomes

The primary outcome measure was knowledge gain. Knowledge was measured before and after the experimental condition for the topics of sleep and social competence. The primary outcome measure was an a priori designed and validated knowledge test consisting of 30 statements (15 per topic) that participants had to answer on a 2-point scale (true or false). Participants received 1 point for each correct answer. Thus, a maximum of 30 points could be achieved in the knowledge test. The internal consistency of the knowledge test was assessed using Cronbach α, which yielded a value of α=0.83, indicating good reliability of the test items. Subsequently, for each statement participants had to report their level of confidence in each response on a scale ranging from 0% to 100%. The outcome score for knowledge gain was the difference in the knowledge test scores before and after the intervention. The experimental condition was either a video or a text (a transcript of the video) or a combination of video and text conveying knowledge about the topics of sleep and social competence (see Experimental Conditions).

### Experimental Conditions

#### Overview

Within the framework of this research project, we developed transdiagnostic videos on sleep and social competence deficits that might serve as psychoeducation material for many disorders (eg, chronic pain or pathological gambling) within the framework of e-mental health programs. The videos were created with the software Videoscribe (Sparkol Limited) and Final Cut Pro X2 (Apple Inc). Participants in the control condition received a 4-min video tutorial of a self-help app with content that was unrelated to sleep or social competence.

#### Video on Sleep

The video on sleep (VS) explained the theoretical framework for the formation and maintenance of sleep disorders, highlighting environmental (eg, noise) and internal (eg, ruminating) maintenance factors. The video explained how disorders are maintained by a learned connection of tension, sleep environment, and stressful thoughts. It also highlighted the negative consequences of sleep deprivation. The total length of the video was 2 minutes, 14 seconds, with a vertical resolution of 1080 pixels and framerate of 15 fps.

#### Text on Sleep

The text on sleep (TS) was a transcript of the video. Thus, the wording was identical in the video and the text.

#### Video on Social Competence

The video on social competence (VSC) explained that social competence is the ability to find a balance between one’s own needs on the one hand and social adaptation on the other. Collectively, 3 different skills are crucial for social competence, all of which can be learned and practiced. The first skill is asserting one’s own interests. The second skill is positive relationship building, such as in a partnership, to protect both one’s own interests and at the same time the interests of the other person. The third skill is the ability to solicit sympathy from other people, such as when contacting strangers or in situations where one wants something from one’s counterpart. The total length of the video was 3 minutes, 57 seconds, with a resolution of 1080 pixels and framerate of 24 fps.

#### Text on Social Competence

The text on social competence (TSC) was a transcript of the video. Thus, the wording was identical in the video and the text.

#### Conditions

The conditions were categorized by sequence (ie, whether the medium alternated or remained the same, that is, video to text vs text to text), topic (sleep vs social competence), and media type (video vs text), resulting in 8 experimental conditions. A control condition was also included in the study. The 8 experimental conditions were as follows:

1. VS, then TSC (n=19)
2. TS, then VSC (n=17)
3. VS, then VSC (n=20)
4. TS, then TSC (n=16)
5. VSC, then TS (n=18)
6. TSC, then VS (n=16)
7. VSC, then VS (n=18)
8. TSC, then TS (n=18)

Condition 9 served as the control condition, consisting of a neutral video stimulus (n=16).

### Statistical Analysis

Data were analyzed using SPSS Statistics (version 29; IBM Corp). To gain a better understanding of knowledge gain in e-mental health programs, multiple statistical analyses were conducted. The analyses aimed to compare the experimental conditions to the control condition and to assess the impact of knowledge deficits on knowledge gain as well as the potential effects of medium, topic, and sequence on knowledge gain. Specifically, to compare knowledge gain between the intervention and control conditions, a 3-way ANOVA considering topic (sleep and social competence) and time (pre- and postintervention knowledge tests) as within-subject factors and condition (experimental vs control) as the between-subject factor was calculated. To assess knowledge gain in individuals with initial deficits in sleep or social competence, a 2-way ANOVA with deficit groups (participants with deficits in sleep, social competence, both, or none) as the between-subject factor and topic as the within-subject factor was performed. To examine the effects of medium, topic, and sequence on learning outcomes, a multivariate ANOVA (MANOVA) with knowledge gain differences as the dependent variables and various independent factors, including medium and deficit group, was applied. Subsequently, to evaluate the impact of knowledge gain on confidence, a 4-way ANOVA using, time, accuracy, and topic as the within-subject factors and condition as the between-subject factor was conducted. These analyses provided insights into factors influencing knowledge gain in the experimental conditions used in this study. An α error of 5% (2-sided) was used for all analyses. As the primary aim of this study was exploratory, parametric tests such as *t* tests, ANOVA, and MANOVA were used. A visual inspection of assumptions did not reveal serious violations that would impact the validity of the analyses.

## Results

**Figure 1:**
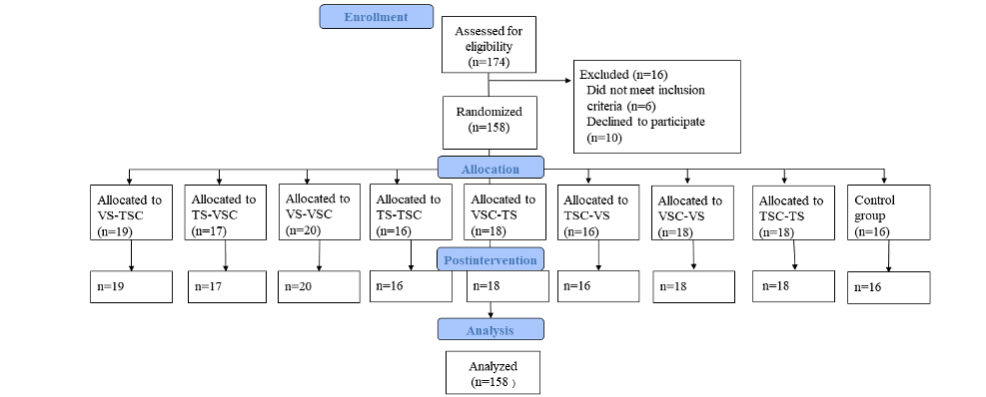
CONSORT (Consolidated Standards of Reporting Trials) flow diagram. TS: text on sleep; TSC: text on social competence; VS: video on sleep; VSC: video on social competence.

Table 1 presents the knowledge score (primary outcome) for all groups, showing the changes in the pre- and postintervention assessments. The flowchart in Figure 1 presents a detailed description of the participant flow through the study.

**Table 1:** Mean knowledge scores before and after interventions for sleep and social competence across 9 experimental conditions in a web-based e-mental health study. Participants were exposed to combinations of video- and text-based psychoeducation or served as controls. The study was conducted online.

	Participants, n	Preintervention score for sleep, mean (SD)	Postintervention score for sleep, mean (SD)	Preintervention score for social competence, mean (SD)	Postintervention score for social competence, mean (SD)
Condition 1: video on sleep, then text on social competence	19	11.47 (1.95)	11.84 (1.61)	8.68 (1.38)	9.32 (1.25)
Condition 2: text on sleep, then video on social competence	17	9.94 (1.71)	10.71 (1.96)	8.59 (1.77)	8.65 (1.87)
Condition 3: video on sleep, then video on social competence	20	9.45 (2.52)	10.10 (2.77)	8.70 (1.17)	8.90 (1.12)
Condition 4: text on sleep, then text on social competence	16	9.81 (2.32)	9.38 (2.45)	8.31 (1.66)	8.81 (1.64)
Condition 5: video on social competence, then text on sleep	18	9.50 (1.86)	10.11 (2.22)	9.22 (1.17)	9.89 (1.23)
Condition 6: text on social competence, then video on sleep	16	11.06 (1.53)	10.94 (2.21)	8.25 (1.53)	8.94 (1.57)
Condition 7: video on social competence, then video on sleep	18	9.78 (2.67)	10.33 (2.83)	8.50 (1.62)	9.17 (1.54)
Condition 8: text on social competence, then text on sleep	18	10.17 (2.26)	10.50 (2.26)	8.50 (1.42)	8.83 (1.29)
Condition 9: control	16	10.00 (2.31)	9.25 (2.32)	8.12 (1.20)	8.88 (1.20)

### Effectiveness of the Experimental Conditions for Knowledge Gain in Comparison to the Control Condition

**Figure 2:**
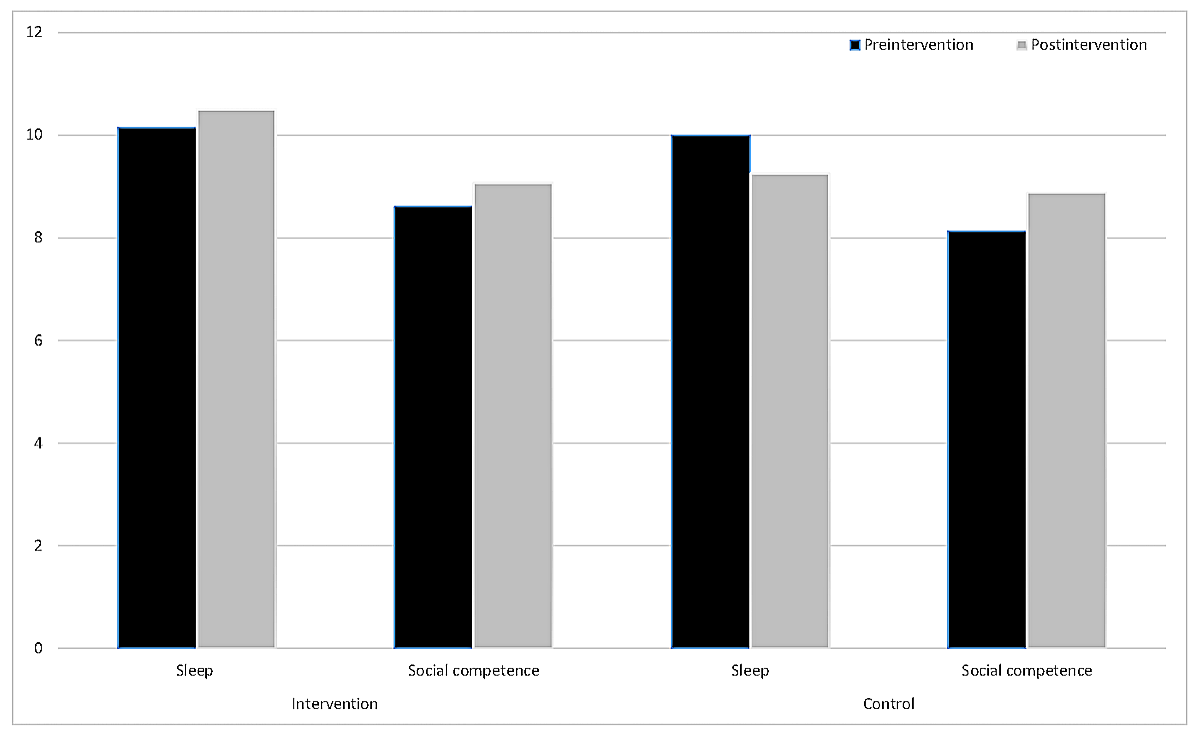
Pre- and postintervention knowledge scores for sleep and social competence in the intervention and control groups. The results demonstrate changes in knowledge levels following the intervention, highlighting differences between the two groups for both topics.

To assess knowledge gain from the first to the second (pre- and postexperimental) knowledge tests, the 8 experimental conditions were pooled into 1 group and compared with the control group (Figure 2). A 3-way ANOVA with domain (sleep and social competence) and time (pre- and postintervention) as the within-subject factors and group (pooled experimental group vs control group) as the between-subject factor was performed. The analysis yielded a significant main effect for time (pre- to postexperimental condition: *F*_1,156_=17.272; *P*<.001; ηp^2^=0.10), indicating that knowledge was successfully increased from before to after the intervention. Furthermore, a significant interaction of topic, time, and condition was observed (*F*_1,156_=6.737; *P*=.01; ηp^2^=0.04), reflecting that the pooled experimental condition improved scores on the knowledge test for both topics (sleep: preintervention mean 10.14, 95% CI 9.78-10.50 and postintervention mean 10.50, 95% CI 10.11-10.89; social competence: preintervention mean 8.61, 95% CI 8.37-8.85 and postintervention mean 9.07, 95% CI 8.83-9.31) across time. In contrast, the control condition did not lead to improvement on the knowledge test on sleep over time (preintervention mean 10.00, 95% CI 8.72-11.28 and postintervention mean 9.25, 95% CI 7.97-10.53). Unexpectedly, the control group showed some improvement on the knowledge test on social competence (preintervention mean 8.13, 95% CI 7.47-8.80 and postintervention mean 8.88, 95% CI 8.22-9.54).

### Knowledge Gain in Participants With Initial Deficits in Sleep and Social Competence

Individuals who reported social competence deficits showed less knowledge about social competence as assessed by the knowledge test at baseline compared to participants without reported deficits in social competence (t_156_=2.12; *P*=.04; *d*=0.41, 95% CI 0.04-1.14). At a statistical trend level, this was also true for individuals with reported sleep deficits (t_156_=1.71-.09; *d*=0.28, 95% CI −1.04 to 0.67).

To further assess knowledge gain in individuals with sleep deficits or social competence deficits, a 2-way ANOVA with the deficit groups (participants with deficits in social competence, deficits in sleep, none, or both) as the between-subject factor and topic (sleep and social competence) as the within-subject factor was performed. Gain in knowledge (from the preintervention to the postintervention knowledge test) served as the dependent variable. A significant main effect for topic was present (*F*_1,154_=4.883-.03; η_p_^2^=0.03), reflecting greater knowledge gain in the social competence knowledge test compared to the sleep knowledge test for all participants. Furthermore, an interaction effect, which bordered on significance, was found for topic and sample (*F*_3,154_=2.210-.09; η_p_^2^=0.04), showing a greater knowledge gain for social competence than for sleep in individuals with prior social competence deficits and a greater knowledge gain for sleep than for social competence in individuals with sleep deficits. Individuals who reported both sleep deficits and social competence deficits or who did not report deficits in either sleep or social competence showed similar knowledge gain for both topics. Post hoc tests showed that those with social competence deficits had significant knowledge gain (t_141_=7.12; *P*<.001; *d*=0.60, 95% CI –0.76 to −0.42) as did those with sleep deficits (t_141_=2.43-.016; *d*=0.20; 95% CI −0.37 to −0.04) from the preintervention to postintervention knowledge tests.

### Examining the Impact of Medium, Sequence, and Topic

To investigate the potential effects of medium, sequence, and topic on learning outcomes, a MANOVA was conducted. Two indices of knowledge gain (ie, differences in knowledge from baseline to postassessment for the topics of sleep and social competence) served as dependent variables. Independent factors were the different sequences of the two media (ie, video to text or text to video), the medium shown first (video or text), topic displayed first (sleep or social competence), use of only 1 medium (ie, video and video, text and text), and deficit group (participants with deficits in sleep, social competence, none, or both). For the omnibus analyses for sleep, none of the main effects or interaction effects achieved significance (*P*>.05). For social competence, subsidiary analyses showed only 1 significant main effect, which was for the medium shown first (*F*_1,157_=4.058-.05; η_p_^2^=0.04), indicating greater knowledge gain when the video was shown first. This result would likely not remain significant after correction for multiple comparisons.

### Effects of Knowledge Gain on Confidence in Correct and Erroneous Items

**Figure 3:**
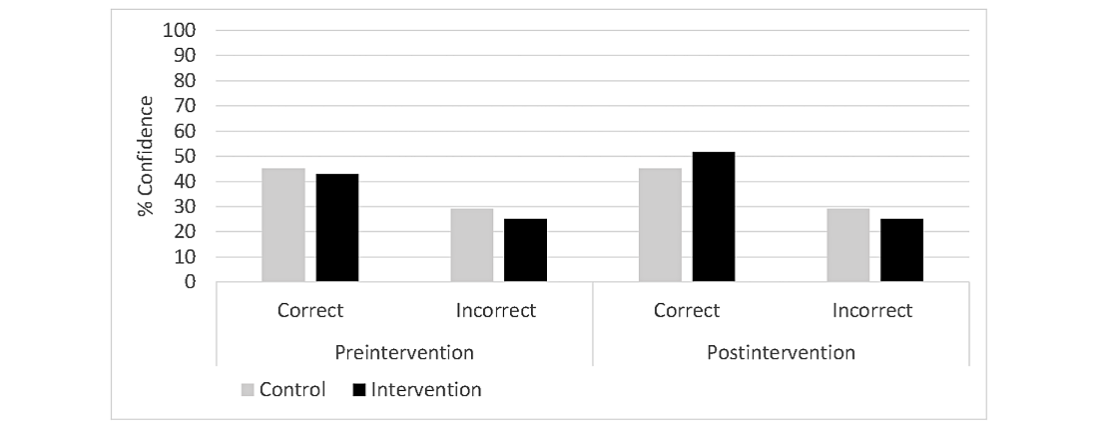
Percentages of correct and incorrect answers before and after the interventions across the control (gray bars) and intervention (black bars) groups. The data highlight changes in confidence levels for correct and incorrect responses, showing the effects of the intervention on participant confidence.

To assess the influence of knowledge gain through video or text on confidence in a knowledge test on the topics of sleep and social competence, a 4-way ANOVA with time (pre- and postintervention knowledge tests), accuracy (correct and incorrect), and topic (sleep and social competence) as the within-subject factors and condition (pooled experimental condition and control condition) as the between-group factor was conducted (Figure 3). Confidence served as the dependent variable. A significant main effect for time was found (*F*_1,157_=9.354; *P*<.001; η_p_^2^=.06), indicating an overall rise in confidence from pre- to postintervention. This was qualified by a significant interaction for time and accuracy (*F*_1,157_=9.354; *P*<.001; η_p_^2^=0.06), suggesting a confidence gain from preintervention (mean 43.95, 95% CI 41.32-46.59) to postintervention (mean 48.32, 95% CI 44.58-52.07) for correct items only; confidence for incorrect items remained unchanged over time (at both times: mean 27.18, 95% CI 24.79-29.58). Additionally, analyses revealed a significant main effect for accuracy (*F*_1,157_=98.735; *P*<.001; η_p_^2^=0.39), indicating higher confidence for correct than incorrect items overall. Another significant interaction emerged for time, accuracy, and group (*F*_1,157_=9.797-.002; η_p_^2^=0.06), indicating a gain in confidence for correct items from preintervention (mean 42.82, 95% CI 41.15-44.50) to postintervention (mean 51.67, 95% CI 49.28-54.04) in the pooled experimental condition, whereas the control condition showed essentially the same confidence from preintervention (correct: mean 45.09, 95% CI 40.09-50.08; incorrect: mean 29.19, 95% CI 24.65-33.73) to postintervention (correct: mean 44.98, 95% CI 37.89-52.08; incorrect: mean 29.19, 95% CI 24.65-33.73) for both correct and incorrect items. Moreover, a significant interaction for time, accuracy, and topic was found (*F*_1,157_=10.213; *P*<.001; η_p_^2^=0.61, suggesting a higher confidence gain for social competence from preintervention (correct: mean 42.86, 95% CI 41.15-44.50) to postintervention (correct: mean 50.27, 95% CI 46.52-54.03), whereas there was essentially no change in scores for sleep from preintervention (correct: mean 45.05, 95% CI 41.57-48.53) to postintervention (correct: mean 46.37, 95% CI 41.81-50.93) for correct items.

## Discussion

### Overview

One of the aims of this study was to examine which medium (video vs text) is most effective in conveying knowledge in e-mental health programs for individuals with and without psychological deficits, using sleep and social competence as example topics. Our primary outcome was knowledge gain, with a particular focus on the interactions between type of medium (video or text), topic (sleep or social competence), and sequence of presentation. We hypothesized that individuals with sleep deficits or social competence deficits would initially have less knowledge on these topics and that the experimental conditions (information provided by video and/or text) would compensate for these knowledge gaps. Additionally, we investigated how increased knowledge affected confidence in judgment.

### Efficacy of the Interventions for Knowledge Gain and Moderators

The results of this study indicate that the experimental conditions (psychoeducational videos and texts) significantly increased participants’ knowledge from before to after the intervention, aligning with our expectations and corroborating previous research that highlights the effectiveness of psychoeducation across various mental disorders, as confirmed by meta-analyses and literature reviews [8-13]. The two media (video and text) led to roughly the same increase in knowledge. In addition, topic, medium, and sequence generally did not impact knowledge gain, with the exception that for the topic of social competence, the gain in knowledge was greater if videos were shown before texts. However, this result would not have withstood correction for multiple comparisons. To the best of our knowledge, no comparable studies have been conducted in an e-mental health setting to date. Preliminary studies, such as those in the field of education involving medical students, have demonstrated that video can have advantages over text, as learning outcomes are better. However, our results do not confirm this finding.

As expected, participants with subjective deficits did have less knowledge on the respective topics at baseline (for sleep, this was only at the statistical trend level). The results thus lend support to the implicit claim of psychoeducation that knowledge deficits are associated with symptoms. Importantly, those who reported deficits in a topic learned more about that topic compared to participants without reported deficits in that topic as a result of the intervention.

### Impact of Confidence on Knowledge Gain

Our results show that confidence in correct answers increased with increasing knowledge. Thus, our low-threshold approach did more than convey knowledge; the participants also became more confident in their correct answers. In line with these results, overall, psychoeducation interventions have been shown to positively impact confidence in various areas, such as childbirth, newborn care, parenting children with autism spectrum disorder, communication skills in health care settings, disaster preparedness, and managing mental disorders [26-28]. Based on previous research, however, we know that the effect can go both ways: increased confidence can also lead to improved learning outcomes [29,30].

### Strengths and Limitations

This study is among the first to explore knowledge gain mechanisms in e-mental health programs and the relationship between knowledge gain and psychological deficits. To ensure the robustness of our findings, we used two different topics to control for the potential confounding variables of knowledge content and topic. Additionally, we measured not only knowledge gain but also confidence levels. Random group allocation and a control condition enhanced the validity of the results. However, our study also had several limitations. First, the recruitment strategy relied on social media, raising concerns about the representativeness of the sample. Moreover, many university students were included in the sample; as a result, the high educational level of our sample compromises the generalizability of our findings to the general population and especially to patient samples, which often have a lower educational level. The high educational level of the sample may have facilitated knowledge acquisition, particularly through text-based interventions, as such participants are likely more familiar with learning in text-dominant formats. In contrast, individuals with lower educational levels may have difficulty understanding text-heavy content, which could reduce the effectiveness of such interventions. Video-based formats might be particularly beneficial for such populations, given videos’ ability to simplify complex concepts and enhance engagement.

### Future Directions

Our findings suggest that the question might not be about determining whether video or text is superior in psychoeducation. Instead, it might be more beneficial to focus on multimedia integration. Combining various media formats could be key to maintaining learner engagement and motivation. The synergy of text, video, and other multimedia elements might enhance the overall knowledge gain more effectively than any single medium alone. This multimodal approach could cater to diverse learning preferences and aid in sustaining attention and interest over prolonged periods. Future studies should explore the impact of multimedia combinations on knowledge gain, especially in the context of psychoeducational content in e-learning platforms. Based on 14 trials, Emmerson and colleagues [31] found that multimedia approaches (including videos) led to increased adherence compared to written or verbal approaches. However, previous studies indicate that to increase adherence, which could not be tested in our single-session design, videos alone might be better [20,31]. Long-term designs are necessary to test the effects of different media on adherence, ensuring sustained engagement and benefit. Additionally, our study’s limited scope compared to comprehensive e-learning programs highlights the need to investigate how media effectiveness varies with content complexity.

E-mental health programs save resources in comparison to conventional therapies by automating interventions (eg, video vs text), without therapist support. Acting as a versatile toolbox, e-mental health programs offer different media formats. Personalizing these programs to individual preferences—such as allowing the user to choose between videos and text—might enhance their effectiveness. For instance, individuals with cognitive impairments might benefit more from videos if they find text-heavy content challenging, while others might prefer reading to avoid the fast pace of videos. Future studies should explore how such preferences influence knowledge gain and symptom improvement to ensure that e-mental health programs are both individualized and effective.

Future research should also examine the impact of various media (video, text, and audio) on symptom improvement in e-mental health interventions. In other words, it still needs to be demonstrated that an increase in knowledge leads to a reduction in symptoms. If true, this would highlight the crucial role of psychoeducation in psychotherapy [7,20].

Considering the strong tie between confidence and knowledge gain, mental health programs could be improved by including strategies to increase confidence, as increased confidence may lead to a decrease in symptoms. Future studies would be helpful to better understand this relationship.

Although the psychoeducational interventions in this study showed promising results in terms of knowledge gain and improved confidence, their implementation in real-world settings may face challenges. For instance, the high educational level of the sample could limit the transferability of the findings to populations with less education, who might require more tailored or simplified formats, such as videos or interactive content. Furthermore, the interventions were tested in a controlled, single-session setting, which does not account for the sustained engagement required in real-world applications. To address these challenges, future implementations could incorporate adaptive learning pathways that adjust content complexity and format based on the user’s educational background or cognitive abilities. Additionally, the interventions could be embedded in existing e-mental health programs. Future research could also benefit from the inclusion of participants with diagnosed mental disorders to improve external validity and the transferability of results to clinical populations.

### Conclusion

E-mental health is essential in modern mental health care. To maximize its potential, research should focus on optimizing e-mental health programs and understanding the mechanisms of success. This study contributes to this goal by investigating the roles of topic, sequence, and medium in knowledge gain. We found no differences in knowledge gain between video and text formats. Interestingly, individuals with self-reported deficits in 1 of the 2 topics did have less knowledge on the topic but also gained more new knowledge on that topic than those without self-reported deficits. Future studies should explore multimedia effects (video, text, and audio) on symptom improvement in clinical intervention studies. Future research should also examine whether individuals’ preferences for different media (eg, video vs text) influence knowledge gain and confidence.

## Appendix

Multimedia Appendix 1 CONSORT-eHEALTH checklist (V 1.6.1).

## Abbreviations

MANOVA: multivariate ANOVA
TS: text on sleep
TSC: text on social competence
VS: video on sleep
VSC: video on social competence

## Data Availability

The anonymized datasets generated and analyzed during this study are available from the corresponding author upon reasonable request. Requests must include a clear statement of purpose and comply with institutional guidelines.

## References

[1] (2022). World mental health report: transforming mental health for all. World Health Organization.

[2] Andrade LH, Alonso J, Mneimneh Z, Wells JE, Al-Hamzawi A, Borges G, Bromet E, Bruffaerts R, de Girolamo G, de Graaf R, Florescu S, Gureje O, Hinkov HR, Hu C, Huang Y, Hwang I, Jin R, Karam EG, Kovess-Masfety V, Levinson D, Matschinger H, O'Neill S, Posada-Villa J, Sagar R, Sampson NA, Sasu C, Stein DJ, Takeshima T, Viana MC, Xavier M, Kessler RC (2014). Barriers to mental health treatment: results from the WHO World Mental Health surveys. Psychol Med.

[3] Knapp M (2003). Hidden costs of mental illness. Br J Psychiatry.

[4] Karyotaki E, Riper H, Twisk J, Hoogendoorn A, Kleiboer A, Mira A, Mackinnon A, Meyer B, Botella C, Littlewood E, Andersson G, Christensen H, Klein JP, Schröder Johanna, Bretón-López Juana, Scheider J, Griffiths K, Farrer L, Huibers MJH, Phillips R, Gilbody S, Moritz S, Berger T, Pop V, Spek V, Cuijpers P (2017). Efficacy of self-guided internet-based cognitive behavioral therapy in the treatment of depressive symptoms: a meta-analysis of individual participant data. JAMA Psychiatry.

[5] Karyotaki E, Efthimiou O, Miguel C, Bermpohl FMG, Furukawa TA, Cuijpers P, Riper H, Patel V, Mira A, Gemmil AW, Yeung AS, Lange A, Williams AD, Mackinnon A, Geraedts A, van Straten A, Meyer B, Björkelund Cecilia, Knaevelsrud C, Beevers CG, Botella C, Strunk DR, Mohr DC, Ebert DD, Kessler D, Richards D, Littlewood E, Forsell E, Feng F, Wang F, Andersson G, Hadjistavropoulos H, Christensen H, Ezawa ID, Choi I, Rosso IM, Klein JP, Shumake J, Garcia-Campayo J, Milgrom J, Smith J, Montero-Marin J, Newby JM, Bretón-López Juana, Schneider J, Vernmark K, Bücker Lara, Sheeber LB, Warmerdam L, Farrer L, Heinrich M, Huibers MJH, Kivi M, Kraepelien M, Forand NR, Pugh N, Lindefors N, Lintvedt O, Zagorscak P, Carlbring P, Phillips R, Johansson R, Kessler RC, Brabyn S, Perini S, Rauch SL, Gilbody S, Moritz S, Berger T, Pop V, Kaldo V, Spek V, Forsell Y, Individual Patient Data Meta-Analyses for Depression (IPDMA-DE) Collaboration (2021). Internet-based cognitive behavioral therapy for depression: a systematic review and individual patient data network meta-analysis. JAMA Psychiatry.

[6] Anderson CM, Hogarty GE, Reiss DJ (1980). Family treatment of adult schizophrenic patients: a psycho-educational approach. Schizophr Bull.

[7] Srivastava Prashant, Panday Rishi (2016). Psychoeducation an effective tool as treatment modality in mental health. Int J Indian Psychol.

[8] Okafor AJ, Monahan M (2023). Effectiveness of psychoeducation on burden among family caregivers of adults with schizophrenia: a systematic review and meta-analysis. Nurs Res Pract.

[9] Powell LA, Parker J, Weighall A, Harpin V (2022). Psychoeducation intervention effectiveness to improve social skills in young people with ADHD: a meta-analysis. J Atten Disord.

[10] Quintiliani MI, Imperatori C, Testani E, Losurdo A, Tamburello S, Contardi A, Della Marca G, Farina B (2020). Usefulness of psychoeducational intervention in chronic insomnia: an actigraphic study. J Ment Health.

[11] Oliveira CTD, Dias ACG (2023). How can psychoeducation help in the treatment of mental disorders?. Estud.Psicol (Campinas).

[12] Brouzos A, Vatkali E, Mavridis D, Vassilopoulos SP, Baourda VC (2021). Psychoeducation for adults with post-traumatic stress symptomatology: a systematic review and meta-analysis. J Contemp Psychother.

[13] Sin J, Gillard S, Spain D, Cornelius V, Chen T, Henderson C (2017). Effectiveness of psychoeducational interventions for family carers of people with psychosis: a systematic review and meta-analysis. Clin Psychol Rev.

[14] Harvey AG, Lee J, Williams J, Hollon SD, Walker MP, Thompson MA, Smith R (2014). Improving outcome of psychosocial treatments by enhancing memory and learning. Perspect Psychol Sci.

[15] Lukens E, McFarlane W (2004). Psychoeducation as evidence-based practice: considerations for practice, research, and policy. Brief Treat Crisis Interv.

[16] Abramowitz JS, Franklin ME, Zoellner LA, DiBernardo Corrie L (2002). Treatment compliance and outcome in obsessive-compulsive disorder. Behav Modif.

[17] Means B, Toyama Y, Murphy R, Bakia M, Jones K (2009). Evaluation of evidence-based practices in online learning: a meta-analysis and review of online learning studies. US Department of Education.

[18] Ebner C, Gegenfurtner A (2019). Learning and satisfaction in webinar, online, and face-to-face instruction: a meta-analysis. Front Educ.

[19] Lin Y, Yu Z (2023). A meta-analysis evaluating the effectiveness of instructional video technologies. Tech Know Learn.

[20] Buch SV, Treschow FP, Svendsen JB, Worm BS (2014). Video- or text-based e-learning when teaching clinical procedures? A randomized controlled trial. Adv Med Educ Pract.

[21] Schlarb AA, Fründ Jan Philipp, Kovacevic T, Faber J (2021). Modularized iCBT‑I self-learn training for university staff-prevention and early intervention in the SARS-CoV-2 crisis: A pilot study. Somnologie (Berl).

[22] Lim SL, Yang J, Ehrisman J, Havrilesky LJ, Reed SD (2020). Are videos or text better for describing attributes in stated-preference surveys?. Patient.

[23] Fischhoff B, Slovic P, Lichtenstein S (1977). Knowing with certainty: the appropriateness of extreme confidence. J Exp Psychol Hum Percept Perform.

[24] Martí L (2021). Certainty and the source of misinformed beliefs [dissertation]. University of California Berkeley.

[25] Dory V, Degryse J, Roex A, Vanpee D (2010). Usable knowledge, hazardous ignorance - beyond the percentage correct score. Med Teach.

[26] Donker T, van Straten Annemieke, Marks I, Cuijpers P (2009). A brief web-based screening questionnaire for common mental disorders: development and validation. J Med Internet Res.

[27] Fenwick J, Toohill J, Slavin V, Creedy DK, Gamble J (2018). Improving psychoeducation for women fearful of childbirth: evaluation of a research translation project. Women Birth.

[28] Shorey S, Chan SW, Chong YS, He H (2015). Perceptions of primiparas on a postnatal psychoeducation programme: the process evaluation. Midwifery.

[29] Magaña Sandy, Lopez K, Salkas K, Iland E, Morales MA, Garcia Torres M, Zeng W, Machalicek W (2020). A randomized waitlist-control group study of a culturally tailored parent education intervention for Latino parents of children with ASD. J Autism Dev Disord.

[30] Cummings CL, Connelly LK (2016). Can nursing students' confidence levels increase with repeated simulation activities?. Nurse Educ Today.

[31] Emmerson KB, Harding KE, Taylor NF (2019). Providing exercise instructions using multimedia may improve adherence but not patient outcomes: a systematic review and meta-analysis. Clin Rehabil.

